# Crumbs and Xpd in mitosis

**DOI:** 10.18632/oncoscience.199

**Published:** 2015-08-20

**Authors:** Eunbyul Yeom, Sung-Tae Hong, Kwang-Wook Choi

**Affiliations:** Department of Biological Sciences, Korea Advanced Institute of Science and Technology, Daejeon, Korea

**Keywords:** crumbs, xpd, mitosis

Mitosis is a fundamental process for chromosome segregation in all multicellular organisms. Misregulation of mitosis can cause genetic instability and cancer. Thus, identification of the genes involved in the regulation of mitosis is important for understanding the mechanism of chromosome segregation and genome instability. Unbiased genetic screens are powerful tools for identifying new gene functions, often resulting in unexpected significant insights. For instance, Human XPD is well-known for its functions in nucleotide excision repair (NER) and transcription [1], but its role in mitosis was suggested from a genetic screen for suppressors of a cell-cycle defect mutant in *Drosophila* [2]. *Drosophila* Xpd identified from this screen was found to be required for the regulation of spindle dynamics and chromosome segregation [3]. Our recent work [4], which identified Galla and Xpd as new partners of Crumbs (Crb) in mitosis, presents another case of fruitful genetic screen.

Crb is a conserved cell membrane protein essential for cell polarity. An unexpected linkage between Crb and mitosis was made through a finding of *galla-1* RNAi as a suppressor of the eye phenotype caused by Crb overexpression. Galla-1 and its paralog Galla-2 turned out to be homologs of human MIP18, a subunit of the MMXD (MIP18-MMS19-XPD) complex [5]. MIP18 localizes with mitotic spindles in growing cancer cell lines and is necessary for proper chromosome segregation [5]. Like MIP18, both Galla proteins co-localize with mitotic spindles in *Drosophila* embryo or S2 cells. *Drosophila* embryo, which undergoes 13 synchronous nuclear division cycles during early stage, offers an excellent system for studying *in vivo* processes of mitosis. RNAi and mutations in *galla* or *crb* cause incomplete nuclear division and abnormal mitotic spindles/centrosomes in early embryos. Furthermore, Galla proteins bind to the intracellular domain of Crb (Crb^intra^), providing compelling evidence for a role of Crb-Galla interaction in mitosis.

As expected from the MIP18-XPD interaction in human cells, Galla physically interacts with Xpd. In addition, Galla and Xpd associate with Crb to form a new Crb-Galla-Xpd (CGX) complex. Genetic evidence suggests that this complex is required for proper chromosome segregation in which Galla and Xpd act downstream to Crb. Because Galla can bind to both Crb and Xpd, it may act as a linker between Crb and Xpd. However, Xpd can also directly interact with Crb. Such multiple modes of interaction may help form a stable complex. Interestingly, despite the similar structure and function of Galla-1 and Galla-2, Xpd preferentially binds to Galla-2. Thus, Galla-2 may be more critical than Galla-1 in forming the CGX complex. It is also possible that Crb and Galla-1 may form a distinct complex with another Xpd-like protein. Like *Drosophila galla* genes, humans have at least two MIP18-related genes, but their functional relationships are unknown.

Mitosis in early embryogenesis leads to nuclear divisions without cytokinesis to generate a multinucleated cell called a syncytial embryo. Thus, it is an important question whether the CGX function is also required for cell proliferation in imaginal discs during organogenesis. Loss of Crb or an increased level of Crb^intra^ induces overproliferation in discs by inhibiting the tumor suppressor function of Hippo (Hpo) [6]. Remarkably, overgrowth of wing disc induced by Crb^intra^ is ameliorated when Galla-1 or Xpd is depleted. This suggests that the CGX function is also required for cell proliferation in disc epithelia, in addition to its role in chromosome segregation in embryo. Intriguingly, hyper-proliferation of wing disc by Crb^intra^ is accompanied by a concomitant increase in DNA damage response to double stranded DNA breaks (DSB) indicated by histone H2Av phosphorylation. The phosphorylated H2Av level is notably decreased by reducing Galla-1 or Xpd expression. Thus, the CGX complex might be involved in the regulation of DNA damage. However, Galla/Xpd effects on the H2Av level might also be an indirect consequence of reduced cell proliferation. Further studies are necessary to delineate the precise role of Crb, Galla and Xpd in cell proliferation and DNA damage control during disc growth.

**Figure 1:**
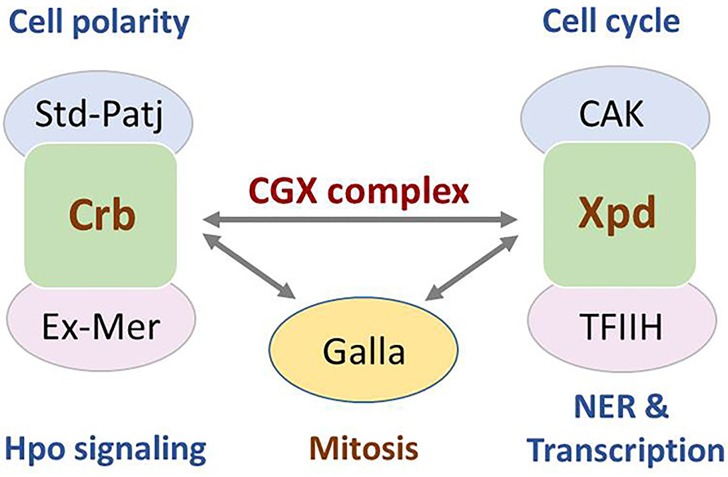
Role of Crb-Galla-Xpd complex in mitosis A scheme of Crb and Xpd complexes involved in distinct functions. Crb forms a complex with Startdust (Std) and Patj to control cell polarity. It also interacts with Expanded (Ex)-Merlin (Mer) to affect Hpo signaling [6]. Xpd in complex with CAK and THIIH regulates cell cycle, NER and transcription [1]. Crb forms a new complex with Galla and Xpd to regulate mitosis [4].

The finding of the CGX complex raises many questions to be addressed in the future. Given the known function of Crb in cell polarity and Hippo signaling [6], it would be interesting to see whether Galla and Xpd may act as regulatory components for proper functioning of Crb and its partners like Expanded in Hippo signaling and Stardust in cell polarity. An important issue in relation to the Crb's role in mitosis is how this transmembrane protein can be localized with the spindle-associated proteins during mitosis. It is possible that Crb might be internalized with endocytic vesicles to interact with Galla and Xpd. MMS19 is another subunit of the MMXD complex that links iron sulfur cluster assembly to DNA metabolism and genomic integrity in mammalian cells [7]. Whether a *Drosophila* MMS19 homolog functions with the CGX complex is an open question. Lastly, mutations in human CRB and XPD genes are intimately associated with genetic diseases, including certain forms of retinitis pigmentosa and xeroderm pigmentosum syndromes. Indeed, defects in mitotic spindles were found in certain mutant cells from XP-D and XP-D/Cockayne syndrome patients [5]. *Drosophila* Xpd mutant alleles associated with high risk cancer also show abnormal centrosomes and cell cycle defects [8]. Therefore, whether and how the CGX complex is related to these diseases is an important topic to be explored further.
